# Unmasking ultradian rhythms in gene expression

**DOI:** 10.1096/fj.201600872R

**Published:** 2016-11-08

**Authors:** Daan R. van der Veen, Menno P. Gerkema

**Affiliations:** * Faculty of Health and Medical Sciences, University of Surrey, Guildford, United Kingdom; and; † Department of Chronobiology, Groningen Institute for Evolutionary Life Sciences, University of Groningen, The Netherlands

**Keywords:** biological rhythm, circadian, metabolism, cell culture, transcriptome

## Abstract

Biological oscillations with an ultradian time scale of 1 to several hours include cycles in behavioral arousal, episodic glucocorticoid release, and gene expression. Ultradian rhythms are thought to have an extrinsic origin because of a perceived absence of ultradian rhythmicity *in vitro* and a lack of known molecular ultradian oscillators. We designed a novel, non–spectral-analysis method of separating ultradian from circadian components and applied it to a published gene expression dataset with an ultradian sampling resolution. Ultradian rhythms in mouse hepatocytes *in vivo* have been published, and we validated our approach using this control by confirming 175 of 323 ultradian genes identified in a prior study and found 862 additional ultradian genes. For the first time, we now report ultradian expression of >900 genes *in vitro*. Sixty genes exhibited ultradian transcriptional rhythmicity, both *in vivo* and *in vitro*, including 5 genes involved in the cell cycle. Within these 60 genes, we identified significant enrichment of specific DNA motifs in the 1000 bp proximal promotor, some of which associate with known transcriptional factors. These findings are in strong support of instrinsically driven ultradian rhythms and expose potential molecular mechanisms and functions underlying ultradian rhythms that remain unknown.—Van der Veen, D. R., Gerkema, M. P. Unmasking ultradian rhythms in gene expression.

Biological rhythms are widespread in behavior and physiology (1), and in past decades, the principal molecular mechanisms driving 24-h rhythms at the cellular level have been identified (2). From this work, it has emerged that these circadian rhythms play a critical role in human health and well-being and that the adverse effects of disrupting biological rhythms include obesity, diabetes, cancer, and mood disorders (3–5). We know considerably less about ultradian rhythmicity, which is a catch-all term for biological rhythms, with periods ranging from milliseconds to hours. Of particular interest to us are ultradian rhythms in the hourly range. Well-known examples of such rhythms include cycles in behavioral arousal (6–9), glucocorticoid level (10), rapid eye movement (REM)–non-REM sleep cycle (11), central monoamine release (12), cellular metabolism (13), and gene expression (14). Despite the broad recognition that these cycles exist, we know nothing about the biological mechanism driving these rhythms and hardly know their functional significance (15).

Ultradian rhythms are prevalent across species, and there are good arguments that they are intrinsically driven and not just imposed by external cycles. Ultradian behavioral locomotor patterns persist under constant environmental conditions (6–8). Experimental deprivation of sleep and food intake strongly suggests an ultradian clock regulation of activity onsets in voles (8). When investigated in mammals, ultradian locomotor rhythmicity is independent of the central circadian clock, and brain substrates such as the retrochiasmatic area of the hypothalamus (16) and the midbrain dopaminergic system (12) have been found to be involved in driving these ultradian patterns. Moreover, ultradian rhythms in *in vitro* cell cultures have been reported for glucocorticoid release (17, 18), single-cell firing (19), and protein synthesis (20) suggesting that these rhythms are intrinsically driven at the cellular level, but mechanisms driving them remain unknown.

So far, a fundamental obstacle in elucidating ultradian mechanisms seems to be the identification of cellular molecular correlates of ultradian rhythms *in vitro*, despite conducive attempts to measure these (14). One of the main reasons for this lack of success may be that ultradian rhythms are often coexpressed with circadian rhythms, which results in ultradian rhythms being overshadowed, or masked by the coexpressed circadian rhythms and their harmonics.

Biological masking of ultradian rhythms is a common phenomenon in behavioral activity: ultradian locomotor patterns, in rodents and *Drosophila*, for example, can be challenging to discern when the same animals also exhibit robust circadian timing (6, 7). When these circadian patterns in behavior are attenuated or removed by means of a surgical (16, 21) or genetic lesion (7, 22–27) of the circadian clock, robust ultradian locomotor rhythms appear. Coexpression of circadian and ultradian rhythms may also be prevalent in gene expression; this notion is supported by the recent finding that *Per1*, *Per2*, and *Bmal1*, all genes that are central to the circadian clock, exhibit both circadian and ultradian expression patterns in the hypothalamus of freely moving rats (28).

We have shown in a prior study that the hepatic expression of these clock genes is associated with both the ultradian and circadian timing system in the vole, a rodent in which the balance between ultradian and circadian timing of behavior can be altered (29). When the vole expresses strong ultradian behavioral patterns, liver expression of these clock genes is flat on a circadian time scale, whereas these same genes exhibit robust circadian expression patterns when the voles show strong circadian timing of behavior when they are housed with a running wheel, or food access is restricted to a 12 h period (30).

These findings confirm the presence of coexpression of ultradian and circadian rhythms in gene expression and suggest that these rhythms are more or less apparent (or more or less masked), depending on the relative contribution of the ultradian timing system to overall biological timing. Moreover, we tend to measure biological rhythms in our experiments on a circadian resolution (*i.e.*, every 3 or 4 h), which captures only the circadian, not the ultradian, timing. We hypothesize that this “parallactic” (31) circadian view, at least in part, underlies our lack of success in detecting ultradian rhythms in gene expression under *in vitro* conditions. An added complication is the common practice in chronobiological signal processing to identify a single (circadian) rhythm and deem higher frequency signals as mathematical harmonics, rather than resolving them as a second (or more) coexpressed rhythm.

To resolve the issues surrounding the hypothesized masked ultradian rhythmicity, we set out to develop a novel analysis pipeline of gene expression that filters out low-frequency, circadian, and stochastic variation in time series of gene expression and relies on analysis methods in the time-, rather than frequency-domain. Using this method on the only publicly available time series of gene expression on an ultradian resolution (14), we for the first time identified expression of *bona fide* ultradian rhythms in gene expression *in vitro*. We showed that both *in vivo* and *in vitro* ultradian gene expression is significantly enriched for metabolic processes and that 60 genes exhibit ultradian expression both *in vivo* and *in vitro*. These 60 genes include genes involved in the cell cycle and are significantly enriched with several DNA motifs in their proximal promotor, which could hold the first clues to unraveling the mechanism that drives ultradian gene expression.

## MATERIALS AND METHODS

We developed a novel 3–criteria-based, non–spectral-analysis pipeline for detecting ultradian rhythmicity, which is based on autocorrelation—a method that lies within the time domain and does not fit harmonics (32, 33). Our approach was used to interrogate data for rhythmicity within a period range of 3–14 h that satisfied 3 *a priori* criteria: the rhythmicity had to be expressed with similar periods throughout the whole dataset; the rhythmicity had to persist after the removal of a low-frequency fundamental signal; and the rhythmic waveform had to be uniformly expressed over all cycles.

**Figure 1.**
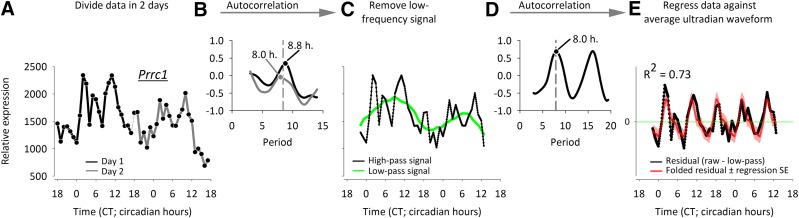
Method of detection of ultradian rhythms in gene expression, with the 48-h expression profile of *Prrc1* used as an example. *A*, *B*) An expression pattern is considered potentially ultradian when both the first and second half of the data (*A*) exhibit similar periodicity in an autocorrelation analysis (*B*). *C*, *D*) The second criterion, that an ultradian rhythm is not an artifact of a low-frequency fundamental signal, is tested by applying a low-pass filter (*C*), and retesting for ultradian periods using autocorrelation analysis (*D*). *E*) Finally, the ultradian waveform must consistently be expressed throughout the dataset, as evidenced by observing a value of *R*^2^ ≥ 0.6 in the regression of the average ultradian waveform against the actual signal.

Our method is graphically described in **Fig. 1**, in the 48-h mRNA expression profile of the proline-rich coiled–coil 1 (*Prrc1*) gene, as reported in the mouse liver by Hughes *et al.* (14). The resolution of the autocorrelation method is limited by the sampling frequency, and, as a first step, we linearly interpolated the dataset to a 0.1-h resolution, for the purpose of obtaining this period resolution.

To test the first criterion that ultradian rhythmicity be consistently evident throughout the time series, we divided the 48-h time series into 2 equal 24-h periods (d 1 and 2; Fig. 1*A*) and used autocorrelation to establish the potential ultradian period (Fig. 1*B*). Any probe that did not exhibit autocorrelation periods in d 1 and 2 that were within 2 h of each other were rejected and not considered for further analysis.

To satisfy the second criterion, that the ultradian rhythmicity be insensitive to removal of low-frequency signals, we applied a low-pass filter on the entire 48-h dataset through boxcar smoothing, with a window size of the average ultradian period of d 1 and 2 (Fig. 1*C*). This step unmasks optional ultradian rhythms and prohibits the occurrence of harmonics of a fundamental circadian signal. The residual signal was reanalyzed by using autocorrelation analysis of the average ultradian waveforms against the data for the whole period (Fig. 1*D*) and was rejected if the most significant period was over 13.5 h. The probe was only accepted if the autocorrelation period of d 1 and 2 and that of the ultradian residual were within 2 h of each other.

The third criterion, which was that the ultradian waveform be consistently expressed throughout the dataset, was tested by establishing the average ultradian waveform by folding the data on that ultradian period (29, 34). This average waveform was nonlinearly regressed against the residual ultradian data, and a cutoff value for the regression value was set. This cutoff was investigated by finding the 95% confidence limit (1.96 sd) of the normal distribution of the regression coefficients (*R*^2^). For the biological data from Hughes *et al.* (14), all potential ultradian signals in the *in* *vivo* liver and *in vitro* fibroblast datasets are shown separately (Supplemental Fig. S1; 0.64 and 0.57 for the liver and fibroblast datasets, respectively), and a combined 95% cutoff of those distributions was set at *R*^2^ ≥ 0.6.

We examined the false-positive rate of our approach by feeding 3 noise datasets, consisting of 45,000 synthetic probes, through our pipeline. All 48 time points for a given probe varied in value within the bounds of an actual biological probe. The 3 noise datasets were dataset 1, white noise: time point values that were randomly varied between the minimum and maximum of a real probe; dataset 2, gaussian white noise: 48 random values that were normally distributed around the mean of a real probe, with a standard deviation of that real probe; and dataset 3, circadian sine combined with 50% gaussian white noise: 48 values describing a circadian sine with random phase and period between 20 and 28 h around the mean and standard deviation of a real probe, where 50% of the variability was gaussian white noise, as in dataset 2.

Our analysis pipeline found 168, 202, and 33 false positives in 45,000 probes in noise datasets 1, 2, and 3, respectively, corresponding to false-positive percentages of 0.37, 0.45, and 0.07%.

The now-established analysis pipeline was then applied to the publicly available 48-h dataset of hourly transcriptome measurements in mouse liver tissue *in vivo* and NIH 3T3 cells *in vitro* that was published by Hughes *et al.* (14). Transcriptome data were downloaded from the Gene Expression Omnibus data repository (35) (GSE11923 and GSE11922, respectively). The *in vivo* mouse liver data were originally acquired by pooling samples of 3 to 5 C57Bl/6J mouse livers on Mouse Genome 430 2.0 Arrays (Affymetrix, Santa Clara, CA, USA). Mice were entrained to a 12 h light, 12 h dark cycle and then released into constant darkness with the first sample taken 18 h after the light–dark cycles was discontinued (which is circadian time 18). The *in vitro* U.S. National Institutes of Health (NIH) 3T3 data were originally acquired from NIH 3T3 cells run on Affymetrix Mouse Genome 430 2.0 Arrays. Circadian rhythms in the cells were synchronized by application of forskolin, and sampling was started 20 h later.

Gene Ontology analysis and Kyoto Encyclopedia of Genes and Genomes (KEGG) pathway analysis of probes that exhibit ultradian expression patterns were performed in WebGestalt (36) using the affy_mouse430_2 array as the background distribution. Motif discovery on the 1000 bp proximal promotor sequences was performed in Meme Suite 4.10.2 (37), in normal mode, searching for motifs between 6 and 50 bp in length.

## RESULTS

**Figure 2.**
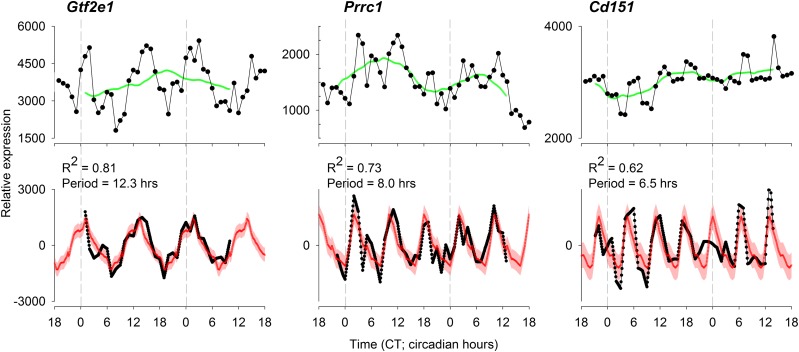
Three examples of probes detected as ultradian by our method. Top: the solid lines show the original expression data obtained from Hughes *et al.* (14), which were not identified as ultradian in their publication. Green line: the low-frequency signal that was removed in our method. Bottom: the achieved residual ultradian signal (plotted in black) and the average ± se ultradian waveform (plotted in red). All 3 probes passed all criteria for ultradian gene expression.

The application of our analysis pipeline on the 48 h time series of gene expression of the mouse liver *in vivo* resulted in a list of 1037 probes that passed all criteria for ultradian gene expression. Of the 323 probes that were identified as ultradian probes in the original publication (14), our method confirmed ultradian expression patterns in 175, exposing a large overlap between both methods, which adds to the validation of our analysis method. **Figure 2** shows ultradian mRNA expression profiles of 3 probes (targeting the murine genes *Gtf2e1*, *Prrc1*, and *Cd151*) as examples of expression profiles that were not previously detected as exhibiting ultradian patterns. We chose 3 examples that exhibit robust ultradian expression patterns with periods of 12.3, 8.0, and 6.5 h—3 periods that are often observed as part of the harmonics of a fundamental circadian signal in frequency domain analysis. These harmonics have been removed in our analysis. *Prrc1* was identified as a circadian probe in the original publication, suggesting that this probe exhibits temporal transcriptional dynamics in both the ultradian and circadian time scale.

**Figure 3.**
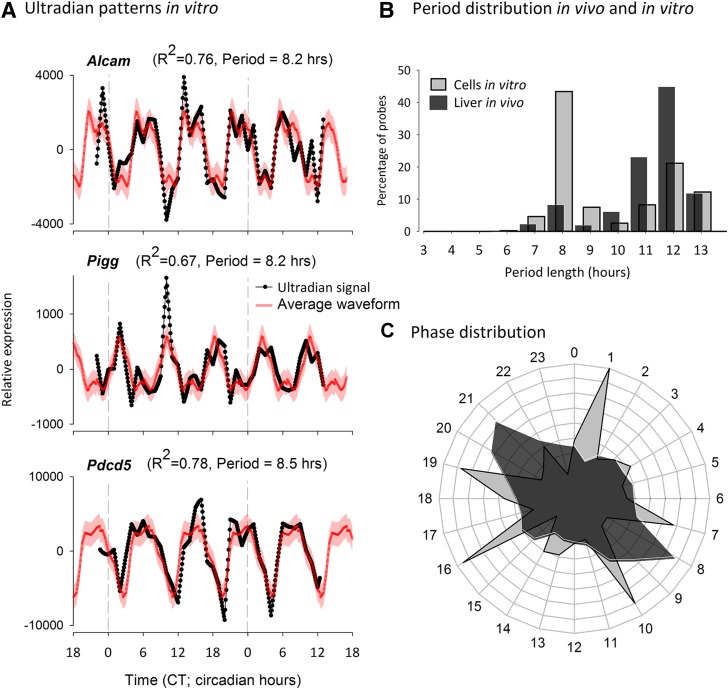
*A*) Examples of ultradian residuals (black) and average ultradian waveform (red) for 3 probes classified as exhibiting ultradian expression patterns in NIH-3T3 cells *in vitro*. *B*) Comparison of the period distributions of ultradian rhythms *in vivo* and *in vitro* demonstrates that the largest group of *in vitro* ultradian rhythms oscillate with a period close to 8–9 h, whereas the largest group of ultradian rhythms *in vivo* exhibit a period close to 12–13 h. *C*) The difference in the period distribution between *in vivo* and *in vitro* ultradian rhythms is also reflected by the clustering of ultradian peak phases, which exhibit more clusters *in vitro* than *in vivo*.

Given the confirmation of our method with previously identified ultradian rhythms in mRNA levels in liver cells *in vivo* and the identification of a substantial number of new ultradian rhythms in gene expression, we next applied our analysis to the ultradian time series of genome-wide gene expression in NIH 3T3 cells *in vitro*, which have been reported not to exhibit ultradian patterns in mRNA expression (14). By contrast to the previous findings, we identified 945 probes that passed all criteria for ultradian gene expression *in vitro*. **Figure 3*A*** depicts 3 examples of probes (targeting murine *Alcam*, *Pigg*, and *Pdcd5*), all exhibiting robust ultradian mRNA expression patterns in NIH 3T3 cells *in vitro*. These 3 examples exhibited rhythmicity within an 8-h period, and we generated a phase distribution plot (Fig 3*B*) that confirmed that a large cohort of our probes showed periods ∼8–9 h in NIH 3T3 cells *in vitro*. Ultradian periods in the *in vivo* murine liver transcriptome dataset also exhibited a cohort of periods of ∼7–8 h, but a larger fraction of probes exhibited periods of ∼12–13 h. This difference in period distribution between *in vivo* liver tissue and *in vitro* NIH 3T3 cells was also reflected in the phase distribution of peaks in mRNA expression profiles, as shown in Fig. 3*C*. Time courses of the low-pass residuals exhibited substantial variation between ultradian probes, with peak expression values occurring throughout the time span (Supplemental Fig. S2).

We next used WebGestalt (36) to examine the Gene Ontology of the lists of probes that express ultradian gene expression in the mouse liver *in vivo* and the list of ultradian genes found in NIH 3T3 cells separately. **Table 1** presents the top 10 categories of both lists and demonstrates a significant enrichment for metabolic process under both conditions, with 5 of 10 enriched processes identical between both conditions.

**TABLE 1.** Top 10 Gene Ontology terms for ultradian genes in both the liver *in vivo* and NIH-3T3 cells *in vitro*

Process	Gene (%)	*P*
*In vivo* liver		
Organic substance metabolic process	41.90	0.0364
Primary metabolic process	40.53	0.0316
Cellular metabolic process	40.21	0.0316
Macromolecule metabolic process	35.03	0.016
Cellular macromolecule metabolic process	32.28	0.0088
Intracellular transport	6.56	0.0316
Protein catabolic process	4.13	0.0316
Proteolysis involved in cellular protein catabolic process	3.07	0.0588
Ubiquitin-dependent protein catabolic process	2.86	0.0588
Intrinsic apoptotic signaling pathway	1.38	0.0588
*In vitro* fibroblasts		
Metabolic process	43.60	5.07E-05
Single-organism metabolic process	40.74	0.0002
Organic substance metabolic process	39.05	0.0006
Cellular metabolic process	38.52	4.98E-05
Primary metabolic process	37.88	0.0003
Macromolecule metabolic process	32.49	0.0002
Cellular macromolecule metabolic process	30.26	5.07E-05
Nucleic acid metabolic process	20.21	0.0006
Chromosome organization	5.40	0.0002
Protein modification by small protein conjugation or removal	4.13	0.0006

With a view to determining common mechanisms and pathways of ultradian gene expression, we identified 28 unique probes that exhibit ultradian gene expression, both in the mouse liver *in vivo* and in NIH 3T3 cells *in vitro*. Furthermore, using the less stringent approach by looking at genes irrespective of probes led to identification of 60 genes that exhibited ultradian mRNA patterns in both *in vivo* and *in vitro* conditions. Although 60 genes is a low number for Gene Ontology analysis, KEGG pathway analysis revealed significant enrichments, which are presented in **Table 2**. Notably, KEGG analysis showed that 3 of 60 genes (*Stag1*, *Ywhae*, and *E2f3*) that are ultradian *in vivo* and *in vitro* are involved in the cell cycle, and we found that a further 2 of the 48 genes (*Terf1* and *Usp28)* are involved in cell cycle checkpoints (38, 39).

**TABLE 2.** Significant KEGG pathways for genes that exhibit ultradian mRNA expression profiles in both *in vivo* and *in vitro* conditions

KEGG pathway	*P*
Cell cycle	0.0318
Base excision repair	0.0318
Non–small-cell lung cancer	0.0426
Glioma	0.0426
Chronic myeloid leukemia	0.0442
ErbB signaling pathway	0.0482
Prostate cancer	0.0482

**Figure 4.**
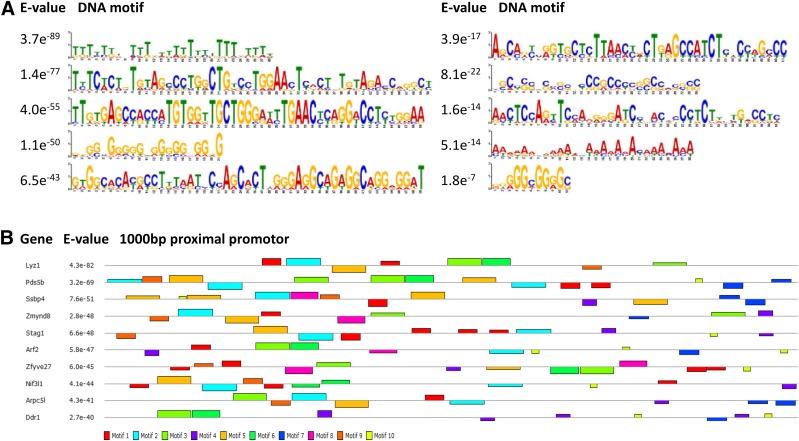
MEME analysis of the 1000-bp proximal promotor of the 60 genes that exhibit ultradian mRNA expression profiles in both *in vivo* and *in vitro* conditions.

As a last step, we submitted the 1000 bp proximal promotor of these 60 genes that exhibit ultradian mRNA expression profiles in both conditions to Multiple EM for Motif Elicitation (MEME) analysis (37) to identify DNA motifs that may be enriched in these proximal promotors and serve as recognition sites for transcription factors. **Figure 4*A*** shows the 10 most significantly enriched DNA motifs, and many of the proximal promotors expressed several of these motifs, of which the most striking examples are given in Fig. 4*B*.

## DISCUSSION

Our approach to unmasking ultradian rhythms for the first time exposed ultradian rhythms in *in vitro* gene expression, a critical line of evidence in strong support of intrinsically driven ultradian rhythmicity that previous attempts could not uncover.

We identified these intrinsically driven ultradian expression patterns in existing datasets generated and published by Hughes and colleagues (14), and the large overlap of ultradian genes identified by them and us in the mouse liver *in vivo* cross-validates our approaches. The identification of ultradian gene expression *in vitro*, where earlier approaches were unsuccessful, is in support of our hypothesis that coexpressed ultradian rhythms (with circadian or other long-term stochastic processes) can be unmasked by filter procedures before signal analysis. In these datasets, the time course trajectories of these long-term processes exhibited substantial variation in expression patterns, which testifies to the varied nature of these masking signals. This finding highlights that unmasking of ultradian rhythms cannot be achieved through application of a single static filter, but only by application of a dynamic filter, which may be a further reason that ultradian rhythms go unnoticed.

Because ultradian rhythms are so diverse, with periods ranging from milliseconds to hours, it is unlikely that they share a common molecular mechanism. Our current focus on rhythms within the hourly range resulted in a diversity of periods across only the 3- to 13-h range. We are acutely aware that the present sampling resolution precluded us from detecting faster rhythms, which for now remains an upcoming challenge. Within our current range, one way forward is to resolve several underlying mechanisms based on clusters of genes within the same period range. Within our period distribution, we saw clear clusters of genes at 4, 8, and 12 h, which cannot be perceived as harmonics of circadian rhythms because our detection methods ruled out mathematical harmonics. We thus showed true biological expression of these ultradian rhythms in gene expression. In terms of causative mechanisms, it is too early to say whether these clusters of rhythms are the result of unique novel mechanism, or even the result of specific coinciding circadian clocks (14). The latter is contrasted, however, by observations of ultradian rhythmicity when circadian clocks are excluded. The observation of these rhythms provides clear validity that some intrinsic mechanism is involved.

Period clustering of different ultradian rhythms, opens the option of mutual coordination or resonance. In terms of functional significance, ultradian rhythmicity is often subjectively associated with metabolic homeostasis *in vivo* (15, 30) and cellular metabolism (13, 40), but these associations are complicated by the difference in ultradian period length. Such a metabolic relevance is objectively supported by the significant enrichment of metabolically relevant genes in our lists of ultradian genes, both *in vivo* and *in vitro*. One consequence of this may be that the plentiful metabolic environment of cells in culture reduces the strength, or robustness of ultradian rhythmicity *in vitro*, in effect causing them to be even more masked by circadian rhythms.

A further clue to the significance of ultradian rhythms in gene expression comes from the specific enrichment of genes associated with the cell cycle. Molecular interactions between the circadian clock and cell cycle checkpoints have been known for some time (41), and although the connection has been made in a completely different species, yeast ultradian cycles have been linked with the cell cycle and metabolism (42). The mammalian cell cycle exhibits a 24-h rhythm, and the molecular circadian clock is proposed to govern daily gating and phase-locking of the cell cycle (43, 44). It has been hypothesized that this circadian timing of the cell cycle serves to protect DNA replication against UV- and ROS-mediated damage (45) and that such rhythms at the cellular level of ROS are strongly associated with circadian rhythms in metabolism (3). The periods of the ultradian genes that we report to be associated with the cell cycle were within the 8.2- to13.2-h range, and one hypothesis may be that they govern the ultradian gating of the cell cycle. Given that we observed a comprehensive enrichment of ultradian gene expression for genes involved in metabolism, such ultradian gating may represent temporal segregation of the DNA replication and metabolism on an ultradian scale, as has also been hypothesized for circadian gating of the cell cycle.

As part of our autocorrelation analysis, we must consider the potential presence of nondeterministic peaks at 1/*f* or 1/*f*^2^. For circadian analysis we often assume that the frequency closest to 24 h is the fundamental signal and discard other frequencies, but for ultradian analysis, the fundamental period is unknown, and we cannot perform such an analysis. For this reason, we reverted to the original time series data, to test our hypothesis based on the autocorrelation analysis. If the hypothesized ultradian period is in fact a spectral alias rather than a deterministic peak in the autocorrelation, the nonlinear regression of the time series data against the ultradian mask should fail to reach a regression coefficient that falls outside the 95% confidence limits of the average regression coefficient. This statistical assumption is corroborated by the results of our (oscillatory) noise models, where the vast majority of the false-positive probes based on autocorrelation analysis failed to reach an *R*^2^ value in the time domain that significantly differed from noise.

If a molecular mechanism can drive biological rhythm, it can be hypothesized that it would do so in both *in vivo* and *in vitro* conditions. Analogous to the circadian timing system, in which it has been shown that, of the only 10 genes that are transcribed with a circadian rhythm in all analyzed tissues, 7 are central to the cellular circadian clock mechanism (46), we found that 60 genes exhibited ultradian gene expression in both *in vivo* and *in vitro* conditions, of which 5 were associated with the cell cycle.

Many analytical tools currently used in the field, such as those that lie within the frequency domain, implicitly assume sinusoidal waveforms. It is important to state that not all our ultradian gene expression patterns exhibited such a sinusoidal waveform. Indeed, examples of nonsinusoidal pulsatile ultradian rhythms have been extensively reported in behavioral activity (6, 7, 16, 21–27), hormone secretion (10), and expression of circadian clock genes, such as *Per1*, *Per2*, and *Bmal1* (28). Such overt, physiological, and molecular pulsatile ultradian rhythmicity is in line with the functional validity of nonsinusoidal ultradian rhythms at the level of gene expression.

It is well established that rhythmic gene expression does not necessarily lead to rhythmic protein abundance (47), and, given the time scale of ultradian rhythmicity and protein stability, it should be established to what extent ultradian rhythms in protein concentration are present and with which physiological and behavioral processes these molecular ultradian rhythms are associated. However, *bona fide* ultradian rhythms in gene expression *in vitro* provide motivation to pursue such links between the molecular generation of ultradian rhythms and the well-known ultradian rhythms in behavior and physiology.

## Supplementary Material

Supplemental Data

## Abbreviations

KEGG: Kyoto Encyclopedia of Genes and Genomes
NIH: U.S. National Institutes of Health
REM: rapid eye movement
