# Supplementary material for: Unmasking ultradian rhythms in gene expression
Source: FASEB J. 2016 Nov 8;31(2):743–50. doi: 10.1096/fj.201600872R (PMC5240665; doi:10.1096/fj.201600872R)
Supplement: Supplemental Data [file supp_31_2_743__index.html]

Unmasking ultradian rhythms in gene expression — Unmasking ultradian rhythms in gene expression — Supplemental Data 

# Unmasking ultradian rhythms in gene expression

## Supplemental Data

- Supplemental Data
- Supplemental Data
