# Supplementary material for: Unmasking ultradian rhythms in gene expression
Source: FASEB J. 2016 Nov 8;31(2):743–50. doi: 10.1096/fj.201600872R (PMC5240665; doi:10.1096/fj.201600872R)
Supplement: Supplemental Data [file supp_fj.201600872R_Supplemental_Figure1.docx]

**Supplemental Figure S1**





Distribution of R^2^ values for all probes with autocorrelation-determined periods within 2 hours of each other on day 1 and day 2 for both the liver *in vivo* and NIH-3T3 cells *in vitro*. The 95% cut-off (mean + 2*SD) of the distributions are 0.64 and 0.57 for the liver and NIH-3T3 datasets respectively, and a combined 95% cut-off of these distributions was set a R^2^ => 0.6.
