# Supplementary material for: Unmasking ultradian rhythms in gene expression
Source: FASEB J. 2016 Nov 8;31(2):743–50. doi: 10.1096/fj.201600872R (PMC5240665; doi:10.1096/fj.201600872R)
Supplement: Supplemental Data [file supp_fj.201600872R_Supplemental_Figure2.docx]

**Supplemental Figure S2**


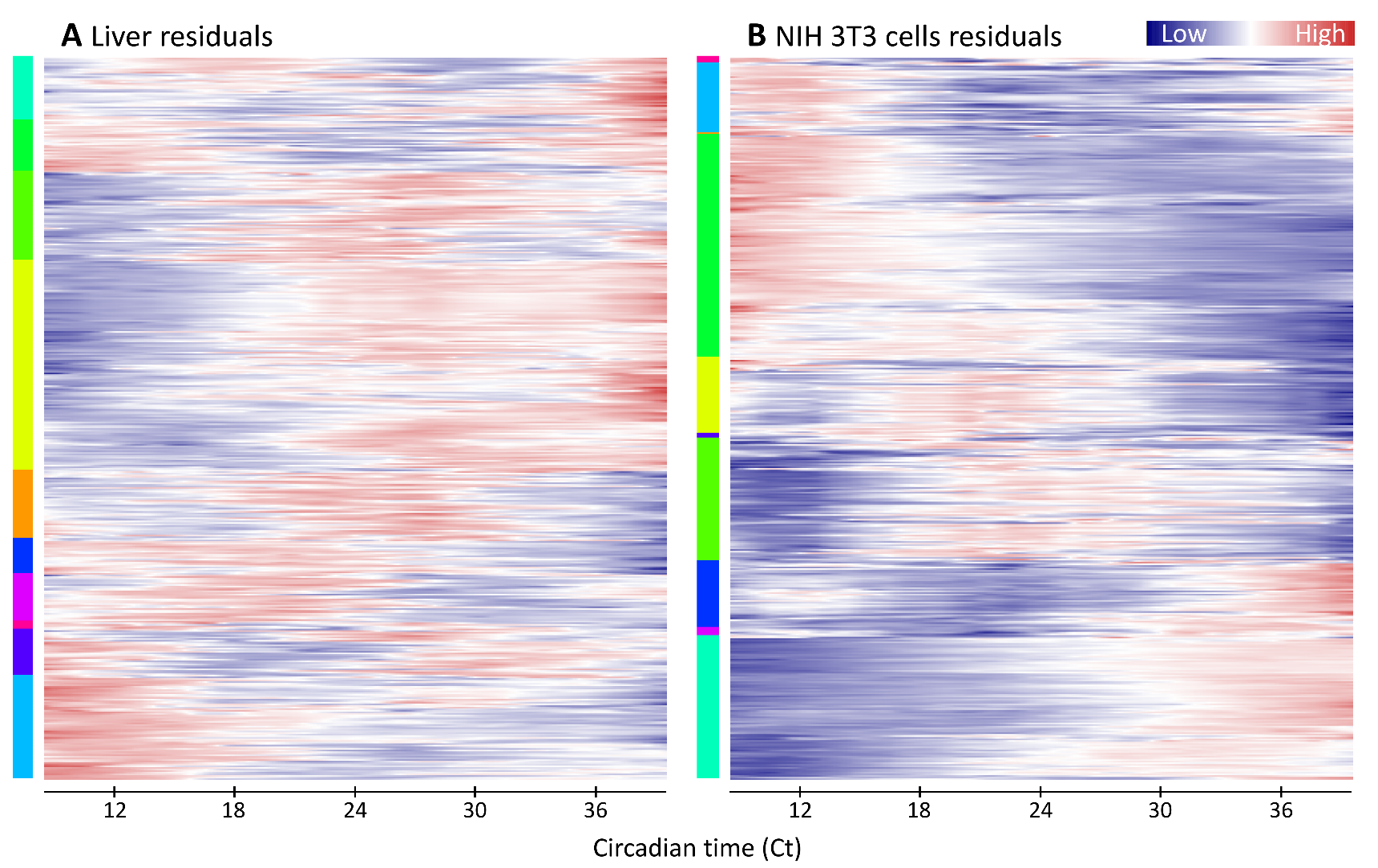


Heatmaps depicting the time courses of all circadian residual signals, which were removed by the low-pass filtering, for probes exhibiting ultradian rhythms in the liver *in vivo* (A) and NIH-3T3 cells *in vitro* (B). The coloured boxes on the left indicate the 10 highest level clusters, as identified in hierarchical clustering based on the temporal dynamics of the time courses. Low-pass filtering truncates the circadian residual signal, and only data from Circadian Time (Ct) 7.5h – 40.5h is included, which was the longest timespan that still included full residuals for all probes.
